# Transcriptomic analysis identifies STAT1 as a potential key transcriptional regulator of T-cell dysfunction in AIHA

**DOI:** 10.3389/fimmu.2026.1896404

**Published:** 2026-10-23

**Authors:** Jing Xu, Linhui Hu, Haixiang Wan, Yixin Yan, Qingming Wang, XueXin Cheng, Zhenjiang Li

**Affiliations:** 1 Department of Hematology, The Second Affiliated Hospital of Nanchang University, Nanchang, Jiangxi, China; 2 Jiangxi Provincial Key Laboratory of Hematological Diseases (2024SSY06052), Nanchang, Jiangxi, China; 3 Jiangxi Province Key Laboratory of Immunology and Inflammation, Jiangxi Provincial Clinical Research Center for Laboratory Medicine, Department of Clinical Laboratory, The Second Affiliated Hospital, Jiangxi Medical College, Nanchang University, Nanchang, Jiangxi, China

**Keywords:** AIHA, CD4+ T cells, CD8+ T cell, STAT1, treatment

## Abstract

**Background:**

Autoimmune hemolytic anemia (AIHA) is characterized by antibody-mediated red blood cell destruction. T-cell dysfunction is implicated in its pathogenesis, but the underlying transcriptional mechanisms remain poorly understood.

**Methods:**

We analyzed peripheral CD4+ and CD8+ T cells from patients with primary warm AIHA and healthy controls by flow cytometry and RNA sequencing. Transcription factor enrichment, ChEA3, an external AIHA Treg dataset, immune-function scores, and clinical correlations were examined. LDH was measured after ex vivo treatment with resveratrol or fludarabine. In an AIHA mouse model, we assessed LDH, spleen weight, erythrocyte-bound IgG, and peripheral T-cell STAT1 and pSTAT1 fluorescence after treatment with resveratrol or fludarabine.

**Results:**

The AIHA group had a higher proportion of peripheral CD8+ T cells and altered CD4+ and CD8+ T cell transcriptional profiles. Transcription factor analysis identified STAT1 as a shared regulatory candidate in both CD4+ and CD8+ T cells. In the mouse experiment, LDH, spleen weight, erythrocyte-bound IgG fluorescence, and peripheral CD4+ and CD8+ T cell STAT1 and pSTAT1 fluorescence were higher in the AIHA group than in controls and lower in the resveratrol and fludarabine groups than in untreated AIHA mice.

**Conclusion:**

Our study identifies STAT1 as a potential transcriptional regulator associated with T-cell dysfunction and hemolytic activity in AIHA. These findings support further investigation of STAT1-associated signaling as a possible therapeutic target.

## Introduction

Autoimmune hemolytic anemia (AIHA) is a heterogeneous group of disorders characterized by autoantibody-mediated destruction of erythrocytes, leading to potentially life-threatening anemia (1). Despite current immunosuppressive therapies, many patients experience refractory disease or treatment-related complications, underscoring the urgent need for more targeted therapeutic strategies (1–3). The pathogenesis of AIHA involves complex immune dysregulation wherein T lymphocytes play crucial roles in supporting autoreactive B-cell responses and directly mediating erythrocyte destruction (4, 5).

However, the molecular mechanisms driving T-cell dysfunction in AIHA remain poorly understood (6), particularly at the transcriptional level. This knowledge gap significantly impedes the development of precision therapies for AIHA. While previous studies have described phenotypic alterations in T-cell subsets (7), a comprehensive analysis of the transcriptomic landscape and its regulatory mechanisms in AIHA T cells is lacking. Identifying key transcriptional regulators could not only advance our understanding of disease pathogenesis but also reveal novel therapeutic targets with direct clinical translation potential.

To address this critical need, our study integrates fundamental mechanistic investigation with clinically relevant functional validation. We aim to transform the understanding and treatment of AIHA. Our findings identify STAT1 as a potential transcriptional regulator associated with T-cell dysfunction in AIHA and provide supportive evidence for its therapeutic targeting, offering a scientifically grounded pathway toward developing more effective and specific treatments for this challenging disorder.

## Methods

### Patients and samples

Between March 2022 and April 2025, peripheral blood was collected from 16 newly diagnosed patients with primary warm autoimmune hemolytic anemia and 16 age- and sex-matched healthy controls. Patients with cold agglutinin disease, mixed AIHA, Evans syndrome, or secondary AIHA were not included. AIHA was diagnosed according to established guidelines (8). Clinical data, including hemoglobin (HB), reticulocyte count and percentage, total bilirubin (TBIL), indirect bilirubin (IBIL), lactate dehydrogenase (LDH), and lymphocyte subset information, were collected at diagnosis before treatment. Additional blood samples from patients in remission and healthy controls were used for ex vivo pharmacological experiments. The study was approved by the Institutional Review Board of the Second Affiliated Hospital of Nanchang University and conducted in accordance with the Declaration of Helsinki. Written informed consent was obtained from all participants or their legal guardians where applicable.

### T cell sorting

Whole blood samples were stained for 30 min at room temperature in the dark using antibodies against CD45-PerCP, CD3-FITC, CD4-PE-Cy7, and CD8-APC-H7 (BD Biosciences, MD, USA). After red blood cell lysis and washing with cold PBS, samples were analyzed on a FACSCanto flow cytometer (BD Biosciences, MD, USA), and data were processed using Diva software.

### RNA extraction, RNA sequencing, and quantitative PCR

CD4+ (5 AIHA and 4 healthy control) and CD8+ T (4 AIHA and 4 healthy control) cells were sorted by flow cytometry from peripheral blood before RNA extraction using TRIzol reagent. RNA integrity was assessed using an Agilent Bioanalyzer, and all samples had RIN values ≥ 7.0. Libraries were prepared and sequenced on an Illumina HiSeq X Ten platform (PE150) by RuiXing Biotechnology Co., Ltd. (Wuhan, China). Raw data were processed using Trimmomatic to remove reads containing poly-N and low-quality reads, yielding 6.71~7.34 Gb of clean data per sample (approximately 25 million paired-end reads per sample). The mapping rate of clean reads to the reference genome ranged from 98.01% to 98.26% across all samples. Clean reads were mapped to GRCh38 with HISAT2; gene-level FPKM values were calculated with Cufflinks and read counts with HTSeq-count. No sample was excluded. All samples were processed in a single batch; batch correction was not applicable. Differential expression was analyzed using DESeq2, with P < 0.05 and |log2 fold change| > 1 as the stated screening thresholds. For quantitative PCR validation, cDNA was analyzed using TB Green (Takara, Japan, catalog no. RR820A) on a Roche LightCycler 480 system (Roche, Basel, Switzerland). GAPDH was the internal control, and relative expression was calculated by the 2^-ΔΔCt method. The primer sequences were as follows: GAPDH-F, GGTCGGAGTCAACGGATTTG; GAPDH-R, GGAAGATGGTGATGGGATTTC; STAT1-F, TTTCCAAAGGCATGGTCTT; STAT1-R, CCTGACATCATTCGCAATT.

### Pathway enrichment and GSEA analysis

Differentially expressed genes from CD4+ and CD8+ T cells were subjected to pathway enrichment analysis. KEGG pathway enrichment was used to identify biological pathways altered in AIHA T cells. Gene set enrichment analysis (GSEA) was further performed using ranked gene lists to evaluate coordinated pathway-level changes between AIHA patients and healthy controls. KEGG enrichment and GSEA were performed with Benjamini-Hochberg FDR correction.

### Transcription factor enrichment and STAT1 target gene analysis

To identify candidate transcription factors underlying AIHA associated transcriptional alterations in T cells, we performed two complementary transcription factor analyses. First, transcription factor enrichment analysis was conducted using transcription factor target gene sets derived from the GSEA database. Ranked gene lists or differentially expressed genes from CD4+ and CD8+ T cells were used to identify transcription factors whose target genes were significantly enriched in AIHA-related transcriptomic changes. Second, ChEA3 was used as an independent transcription factor prediction platform to infer upstream regulators of the differentially expressed genes in CD4+ and CD8+ T cells. Candidate transcription factors identified by both approaches and shared between CD4+ and CD8+ T cells were considered robust candidate regulators. Thirdly, the external Treg RNA-seq dataset GSE195791, reported by Ciudad et al. (7) consisted of sorted Treg cells from patients with primary warm AIHA and age- and sex-matched healthy controls. This dataset was analyzed to determine whether STAT1-associated transcriptional signals identified in CD4^+^ and CD8^+^ T cells were also present in an independent AIHA-derived Treg population involved in immune regulation. Finally, these intersecting transcription factors were further evaluated for differential expression in RNA-seq data and validated where applicable by quantitative PCR.

### Ex vivo STAT1 inhibition and LDH assay

To functionally evaluate the therapeutic relevance of STAT1 inhibition, whole blood samples from newly diagnosed AIHA patients, AIHA patients in remission, and healthy controls (n=3 per group). Fresh peripheral blood samples were collected into sodium heparin anticoagulant tubes, and drug treatment was initiated within 30 minutes of collection. Undiluted whole blood was treated with fludarabine or resveratrol (1, 10, and 100 μM) and incubated for 24 hours at 37 °C in a humidified atmosphere containing 5% CO_2_. Following incubation, samples were centrifuged at 300 × g for 5 minutes, and the supernatant was collected for LDH measurement.

Fludarabine was purchased from Sigma and resveratrol were purchased from Solarbio, LDH levels were measured using an LDH assay kit (Beyotime Institute of Biotechnology, China; catalog no. C0038) according to the manufacturer’s protocol. Each *in vitro* experiment was performed in triplicate.

### AIHA mouse model

Twenty male C57BL/6J mice, aged six weeks, were obtained from Vitalstar Biotechnology (Beijing, China) and housed at the Animal Center of Nanchang University at 23–25 °C. After four days of acclimatization, the mice were randomly allocated to a control group, an AIHA group, an AIHA plus resveratrol group, and an AIHA plus fludarabine group (n = 5 per group). The three AIHA groups received 2 × 10^8^ rat red blood cells (Shanghai Yuanye Bio-Technology, Shanghai, China) in 200 μL sterile saline by intraperitoneal injection once weekly for nine weeks. Controls received 200 μL sterile saline on the same schedule. Starting at week 6 and continuing through the nine-week modeling period, mice received intraperitoneal resveratrol (20 mg/kg daily; MCE, China) or fludarabine (5 mg/kg every three days; Sigma, USA), as assigned.

Freshly isolated erythrocytes were washed three times in cold PBS and stained for 30 min in the dark at room temperature with PE-conjugated anti-IgG antibody (BioLegend, San Diego, CA, USA). Erythrocyte-bound IgG was quantified by mean fluorescence intensity (MFI). For T-cell analysis, cells were surface-stained for CD3, CD4, and CD8; fixed and permeabilized with FOXP3 Fixation/Permeabilization Buffer (BioLegend); and stained for total STAT1 (Proteintech, China) and p-STAT1 (Tyr701; BD Biosciences, MD, USA). CD3+CD4+ and CD3+CD8+ cells were gated as CD4+ and CD8+ T cells, respectively, and STAT1 and pSTAT1 were quantified by MFI. Data were acquired on a BD FACSCanto and analyzed with BD FACSDiva and FlowJo.

### Statistical analysis

Continuous data were summarized as mean ± SD when appropriate for their distribution; otherwise, median (interquartile range) was used. Two-group comparisons used Student’s t test or the Mann–Whitney U test, as appropriate. Multiple-group comparisons used ANOVA followed by the LSD test, as originally reported. Pearson correlation was used for associations between T-cell measures, STAT1 expression, GSVA scores, and clinical indicators. Statistical analyses used GraphPad Prism 5.0 and R 4.0.2. P < 0.05 was considered statistically significant.

## Results

### Peripheral T-cell alterations and clinical correlations in AIHA

We first analyzed peripheral T-cell subset distribution in 16 newly diagnosed AIHA patients and 16 age- and sex-matched healthy controls. The clinical characteristics of the AIHA cohort are summarized in **Table 1**. Flow cytometry showed that the proportion of CD8+ T cells was significantly increased in AIHA patients compared with healthy controls, whereas the proportion of CD4+ T cells did not differ significantly between the two groups (**Figure 1A**).

**Table 1:** Characteristics of AIHA patients.

Characteristic	N = 16^1^
Age, years, mean (SD)	59.56 (17.49)
HB, g/L, mean (SD)	64.56 (10.01)
Ret%, median (IQR)	18.42 (12.20–31.24)
Ret, ×10^9^/L, median (IQR)	342.00 (234.85–496.68)
TBIL, μmol/L, mean (SD)	60.90 (27.62)
IBIL, μmol/L, mean (SD)	48.48 (21.07)
LDH, U/L, median (IQR)	426.04 (303.51–648.05)
Ly, %, mean (SD)	13.62 (7.45)
T/Ly, %, mean (SD)	58.79 (15.43)
CD4T/T, %, mean (SD)	46.70 (16.40)
CD8T/T, %, mean (SD)	42.71 (14.03)
CD4/CD8, median (IQR)	1.22 (0.43–2.27)

Data are presented as mean (SD) for normally distributed variables and median (IQR) for non-normally distributed variables, as determined by the Shapiro-Wilk test.

HB, hemoglobin; Ret, reticulocyte; Ret%, reticulocyte percentage; TBIL, total bilirubin; IBIL, indirect bilirubin; LDH, lactate dehydrogenase; Ly, lymphocyte; T/Ly, T cells/lymphocytes; CD4T/T, CD4+ T cells/T cells; CD8T/T, CD8+ T cells/T cells; CD4/CD8, CD4+ T cells/CD8+ T cells.

**Figure 1:**
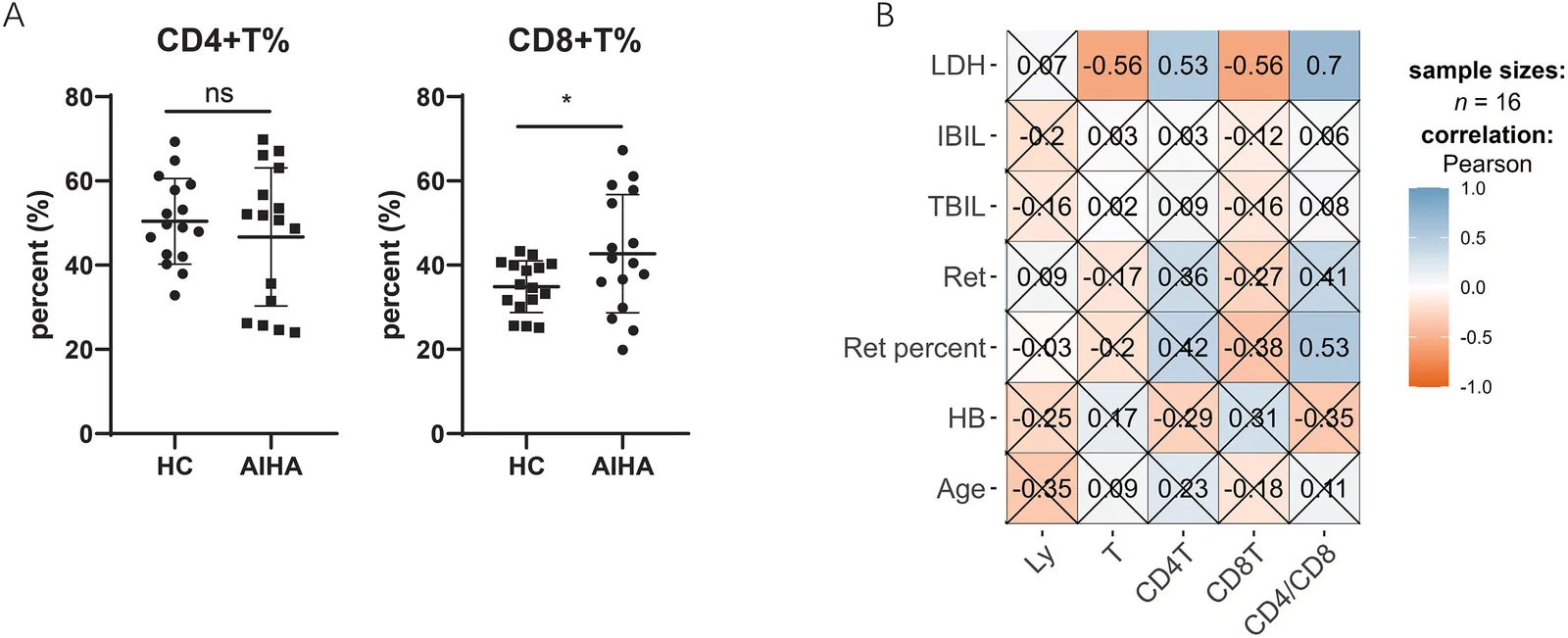
T-cell subset distribution and correlation with clinical parameters in AIHA patients vs. healthy controls. **(A)** Flow-cytometric analysis of peripheral blood CD4+ and CD8+ T-cell proportions in 16 newly diagnosed AIHA patients and 16 age- and sex-matched healthy controls. Data are shown as individual values with mean ± SD. Statistical significance was determined by two-tailed unpaired Student’s t-test (ns, not significant; *P < 0.05). **(B)** Pearson correlation matrix showing associations between CD4+ T-cell proportion, CD8+ T-cell proportion, and clinical parameters, including lactate dehydrogenase (LDH), indirect bilirubin (IBIL), total bilirubin (TBIL), reticulocytes (Ret), and hemoglobin (HB), in AIHA patients (n = 16). Correlation coefficients are shown in each cell. * indicates P < 0.05; non-significant correlations are marked with”X”.

We next examined the relationship between T-cell subset distribution and hemolysis-related clinical parameters in AIHA patients. Correlation analysis showed that CD4+ and CD8+ T-cell proportions were associated with clinical indicators of disease activity, including LDH and bilirubin-related parameters (**Figure 1B**). These findings indicate that peripheral T-cell imbalance, particularly expansion of the CD8+ T-cell compartment, is linked to the clinical immune-hemolytic state of AIHA.

### Transcriptomic remodeling of CD4+ and CD8+ T cells in AIHA

To explore the molecular basis of T-cell dysregulation in AIHA, we performed RNA sequencing of sorted CD4+ T cells from AIHA patients and healthy controls and sorted CD8+ T cells from AIHA patients and healthy controls. Pathway enrichment analysis revealed marked transcriptomic remodeling in both T-cell subsets. In CD4+ T cells, KEGG analysis included cell cycle, cellular senescence, oocyte meiosis, and immune-related pathways (**Figure 2A**). GSEA showed higher enrichment of B-cell receptor signaling, systemic lupus erythematosus, and cell-cycle pathways and lower enrichment of ribosome pathways in AIHA than in controls (**Figure 2B**).

**Figure 2:**
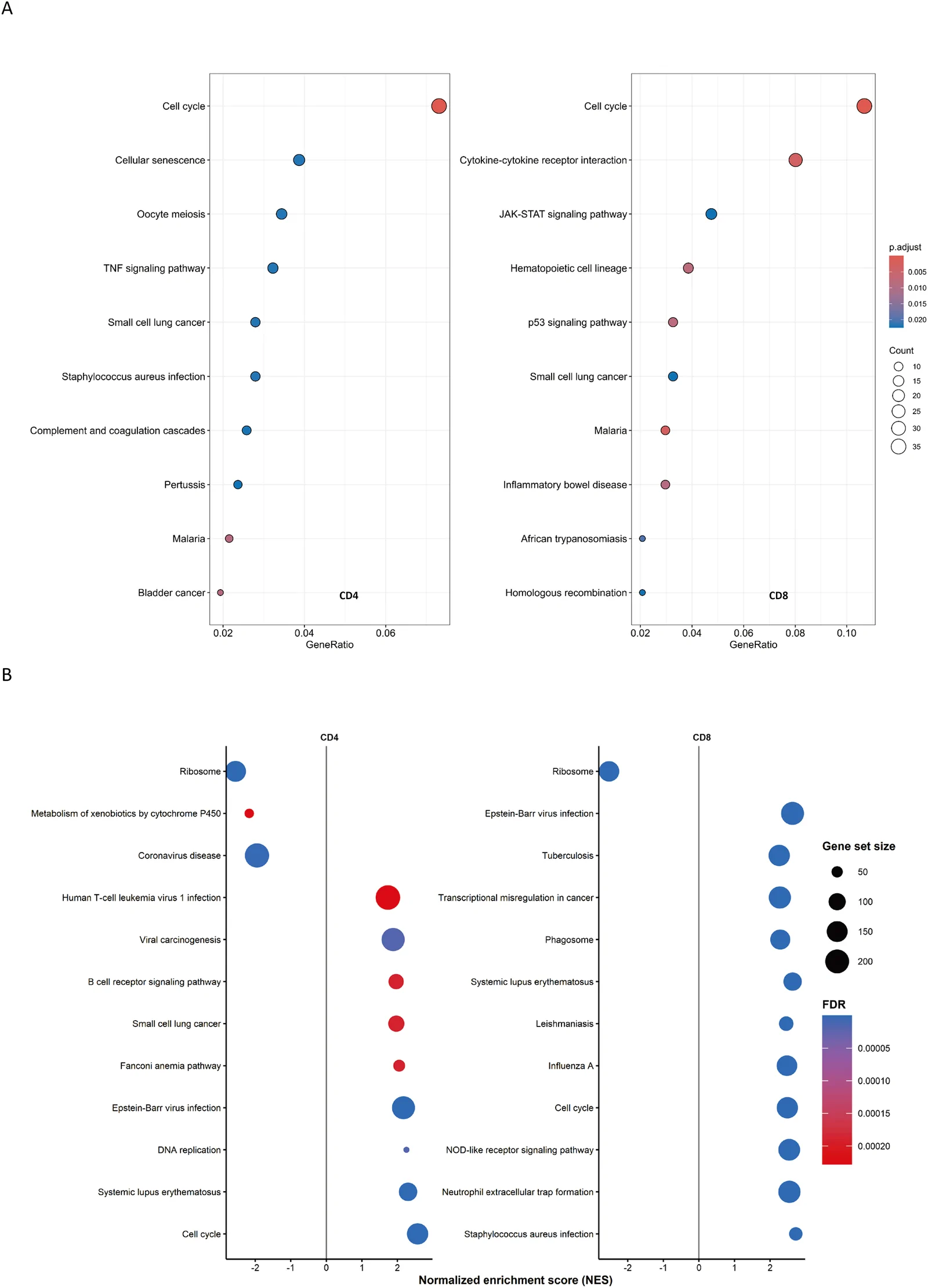
Pathway analyses of sorted CD4+ and CD8+ T cells in primary warm AIHA. **(A)** KEGG enrichment of differentially expressed genes in CD4+ and CD8+ T cells from AIHA patients versus healthy controls. Dot size represents gene count, and dot color represents adjusted P value. **(B)** GSEA of ranked gene-expression changes in CD4+ and CD8+ T cells. Bars represent normalized enrichment scores (NES); positive and negative NES indicate enrichment in AIHA and healthy control samples, respectively. Dot size represents gene set size, and color represents FDR. For both panels, n = 5 AIHA and n = 4 healthy controls for CD4+ T cells, and n = 4 AIHA and n = 4 healthy controls for CD8+ T cells.

In CD8+ T cells, KEGG analysis included cell-cycle, JAK–STAT, and inflammatory or immune-related pathways (**Figure 2A**). GSEA showed higher enrichment of cell-cycle and systemic lupus erythematosus pathways and lower enrichment of ribosome pathways in AIHA than in controls (**Figure 2B**).

### STAT1 is identified as a shared transcriptional regulator in AIHA T cells

Because transcription factors can drive coordinated gene-expression changes, we next performed two complementary transcription factor screening analyses to identify candidate transcription factors in AIHA T cells. First, transcription factor enrichment analysis based on GSEA database-derived transcription factor target gene sets was performed in CD4+ and CD8+ T cells. In parallel, we used ChEA3 to independently predict transcription factors responsible for the differentially expressed genes in CD4+ and CD8+ T cells. To increase the robustness of candidate selection and avoid reliance on a single database or algorithm, we focused on transcription factors that were consistently identified by both approaches and shared by CD4+ and CD8+ T cells.

By intersecting the results from GSEA-based transcription factor enrichment and ChEA3 prediction, STAT1 and FOXM1 were identified by both transcription factor screening approaches in CD4+ and CD8+ T cells (**Figure 3A**; **Supplementary Figures 1A, B**). Notably, in the external AIHA Treg dataset GSE195791, STAT1, not FOXM1 was the top-ranked candidate transcription factor in the ENCODE ChIP-seq library analysis, suggesting enrichment of STAT1 target genes among transcriptional alterations associated with AIHA Treg cells (**Supplementary Figure 1C**). qPCR further supported the increased expression of STAT1 in AIHA T cells.(**Figure 3B**); Therefore, STAT1 was prioritized for subsequent analyses as a shared and externally supported transcriptional regulator of AIHA-associated T-cell dysregulation.

**Figure 3:**
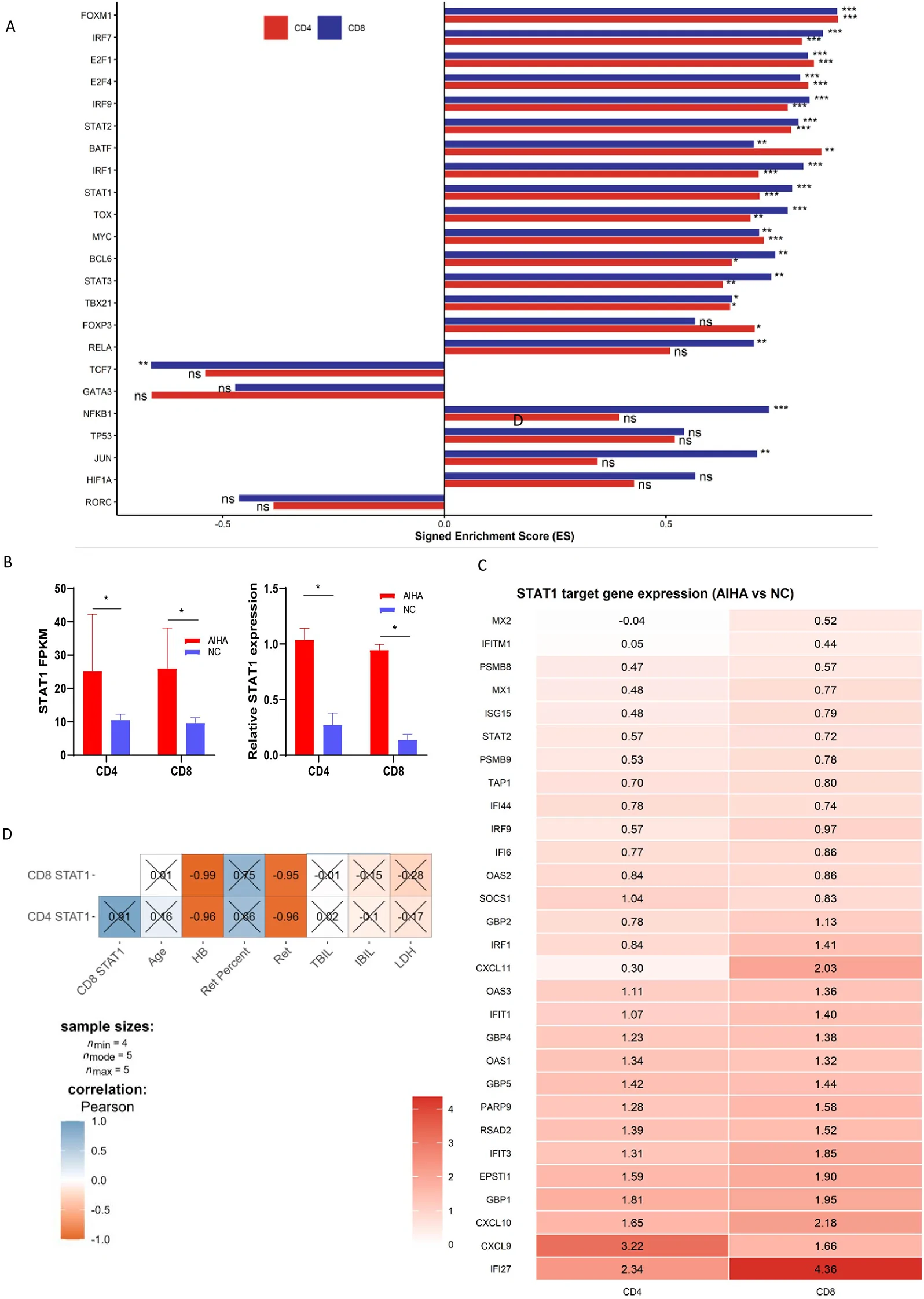
Candidate transcription factors and STAT1-associated measurements in primary warm AIHA. **(A)** Transcription factor target-gene enrichment in CD4+ and CD8+ T cells. Bars represent signed enrichment scores (ES); positive and negative values indicate enrichment in AIHA and healthy control samples, respectively. Red, CD4+ T cells; blue, CD8+ T cells. *P < 0.05, **P < 0.01, ***P < 0.001; ns, not significant. **(B)** STAT1 expression in CD4+ and CD8+ T cells from AIHA patients and healthy controls. Left, STAT1 FPKM; right, relative STAT1 expression. Data are shown as mean ± SD. Statistical significance was determined by two-tailed unpaired Student’s t-test, * indicates P < 0.05. **(C)** STAT1 target-gene expression in CD4+ and CD8+ T cells (AIHA vs. NC). Values represent expression changes; redder color indicates higher expression in AIHA. **(D)** Pearson correlation matrix showing associations between CD4+ STAT1, CD8+ STAT1, and clinical parameters including age, hemoglobin (HB), reticulocyte percentage (Ret Percent), reticulocytes (Ret), total bilirubin (TBIL), indirect bilirubin (IBIL), and lactate dehydrogenase (LDH). Correlation coefficients are shown in each cell; * indicates P < 0.05; non-significant correlations are marked with “X”.

### STAT1 target-gene expression and correlations with immune scores and clinical measures

To further evaluate the downstream transcriptional program associated with STAT1, we analyzed STAT1 target genes in AIHA T cells. STAT1 target genes showed altered expression patterns in both CD4+ and CD8+ T cells from AIHA patients compared with healthy controls (**Figure 3C**), indicating activation of a STAT1-associated transcriptional program in AIHA T cells.

In addition, correlation analysis between STAT1 expression and clinical parameters showed associations between STAT1 levels in CD4+ or CD8+ T cells and hemolysis-related indicators, including HB, reticulocyte parameters, LDH, TBIL, and IBIL (**Figure 3D**). These findings support a relationship between STAT1-associated T-cell dysregulation and clinical disease activity in AIHA.

### Pharmacological STAT1 inhibition reduces LDH levels in active AIHA samples

We assessed whether pharmacological STAT1 inhibition could influence hemolysis-related activity *in vitro*. Whole blood samples from newly diagnosed AIHA patients, patients in remission, and healthy controls were treated with fludarabine or resveratrol for 24 h, and LDH levels were measured as a readout of hemolytic activity. In samples from newly diagnosed AIHA patients, both fludarabine and resveratrol reduced LDH levels in a concentration-dependent or dose-responsive manner (**Figures 4A–D**). In contrast, LDH levels were not significantly altered in samples from remission AIHA patients or healthy controls. These results suggest that pharmacological inhibition of STAT1-related activity can reduce hemolysis-associated LDH release in active AIHA, supporting the therapeutic relevance of STAT1 in a disease-active context.

**Figure 4:**
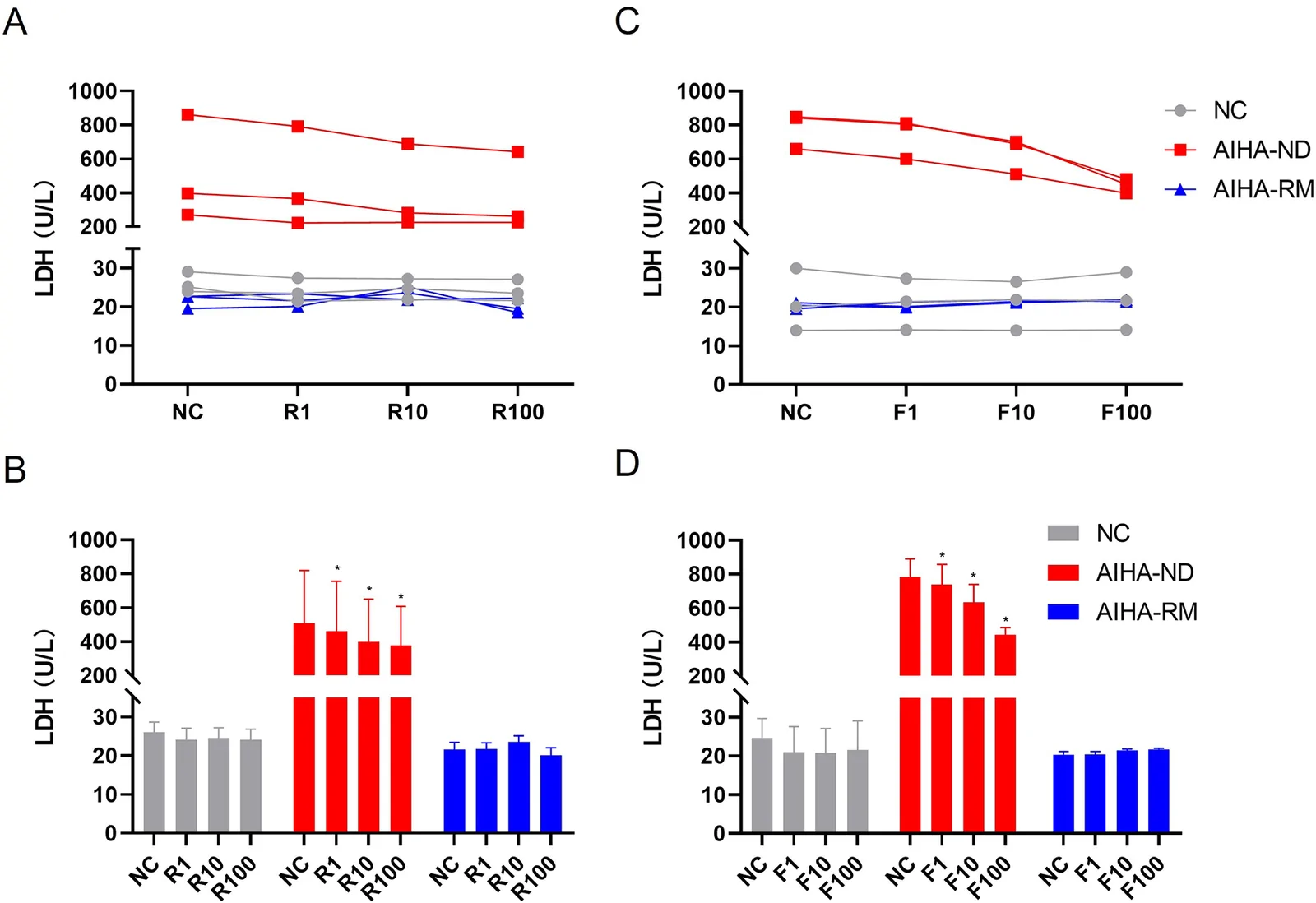
Ex vivo LDH measurements after pharmacological treatment of whole blood. Samples from newly diagnosed patients with AIHA (AIHA-ND), patients in remission (AIHA-RM), and healthy controls (NC) were treated with resveratrol (R) or fludarabine **(F)** at 1, 10, or 100 μM for 24 h. **(A, C)** Individual LDH values across treatment concentrations. **(B, D)** Summary bar graphs of LDH levels. Data are shown as mean ± SD. * indicates P < 0.05.

### Pharmacological inhibition of STAT1 attenuates hemolysis and suppresses STAT1 activation in an AIHA mouse model

Further, we established an AIHA mouse model and evaluated the effects of pharmacological STAT1 inhibition (**Figure 5A**). Twenty male C57BL/6J mice were allocated to four groups (n = 5 per group). Mean erythrocyte-bound IgG fluorescence was higher in the AIHA group than that in controls (**Figure 5B**). Compared with controls, the untreated AIHA group had higher mean serum LDH and spleen weight (**Figure 5B**).

**Figure 5:**
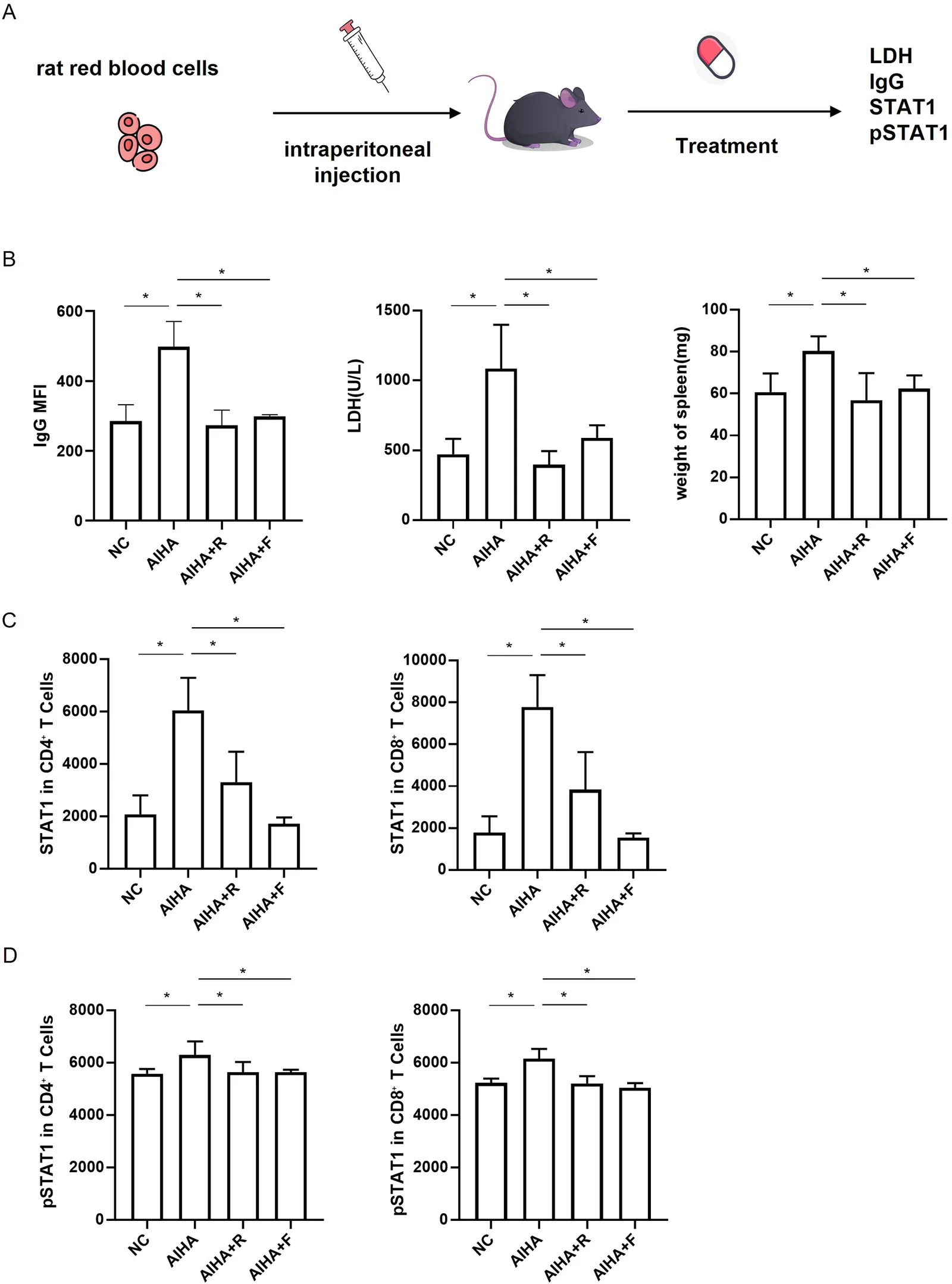
Pharmacological treatment in an AIHA mouse model. **(A)** Schematic of the AIHA mouse model and treatment protocol. Mice received intraperitoneal injections of rat red blood cells to induce AIHA, followed by treatment with resveratrol or fludarabine. LDH, IgG, STAT1, and pSTAT1 were subsequently measured. **(B)** Erythrocyte-bound IgG mean fluorescence intensity (MFI), LDH levels, and spleen weight in control (NC), untreated AIHA, resveratrol-treated AIHA (AIHA+R), and fludarabine-treated AIHA (AIHA+F) mice. **(C)** STAT1 fluorescence in peripheral blood CD4+ and CD8+ T cells. **(D)** Phosphorylated STAT1 (pSTAT1) fluorescence in peripheral blood CD4+ and CD8+ T cells. For all panels, n = 5 mice per group. Data are shown as mean ± SD. Statistical significance was determined by one-way ANOVA with Tukey’s *post hoc* test for multiple comparisons (*P < 0.05). Error bars represent SD.

We next evaluated whether STAT1 signaling was activated during autoimmune hemolysis. Flow cytometric analysis demonstrated that STAT1 expression was increased in peripheral blood CD4^+^ and CD8^+^ T cells from AIHA mice compared with controls (**Figure 5C**). Furthermore, pSTAT1, representing active STAT1 signaling, was also elevated in both T-cell subsets, suggesting enhanced STAT1 pathway activation during AIHA development (**Figure 5D**).

Notably, both resveratrol and fludarabine reduced serum LDH levels compared with untreated AIHA mice, suggesting attenuation of hemolytic activity. Importantly, STAT1 inhibition was accompanied by reduced STAT1 expression and decreased pSTAT1 levels in peripheral blood CD4^+^ and CD8^+^ T cells (**Figures 5C, D**).

Collectively, these findings demonstrate that STAT1 activation accompanies autoimmune hemolysis and that pharmacological inhibition of STAT1 signaling reduces hemolytic activity *in vivo*, providing additional functional evidence supporting STAT1 as a potential therapeutic target in AIHA.

## Discussion

The management of AIHA remains a significant clinical challenge, with current strategies primarily reliant on corticosteroids and rituximab (1). A substantial number of patients experience inadequate responses, relapse, or intolerance to these therapies, highlighting the urgent need for novel treatment targets. Our study bridges this translational gap by identifying STAT1 as a potential therapeutic target in AIHA, supported by integrated transcriptomic and functional evidence.

In recent years, relevant literature studies have found that in the complex pathogenesis of AIHA, abnormal regulation of CD4+ and CD8+ T cells plays a crucial role, specifically manifested as a decrease in the number of CD4+ T cells and abnormal clonal proliferation of CD8+ T cells, which are closely associated with the occurrence and development of AIHA (6, 9, 10). We also observed a higher CD8+ T-cell proportion in affected patients and cell-cycle pathway enrichment in sorted CD8+ T cells. This pattern is consistent with changes in the CD8+ compartment, although pathway enrichment alone does not demonstrate clonal expansion. The correlations of T-cell measures with clinical indicators in this small cohort should be interpreted as associations.

The relevance of the external Treg analysis is supported by previous evidence that Treg abnormalities contribute to impaired immune regulation in warm AIHA, as reported by Ciudad et al. (7) The identification of STAT1 target-gene enrichment in an independent AIHA-derived Treg dataset provides complementary support for the involvement of STAT1-associated transcriptional regulation across immune-regulatory T-cell populations. However, this enrichment does not establish a causal role for STAT1 in Treg dysfunction, prove STAT1 activity in Treg cells, or demonstrate identical regulatory mechanisms among different T-cell subsets. The limited sample size of the external dataset and differences in T-cell populations analyzed should also be considered when interpreting these findings.

The ex vivo whole-blood experiments showed that resveratrol and fludarabine exposure reduced LDH levels, consistent with a decrease in hemolysis-related activity. Because LDH is released during hemolysis and the whole-blood assay preserves the interaction between T cells and other blood components, these results support a treatment-associated reduction in hemolytic activity in a setting relevant to AIHA (11). The parallel reduction in LDH and STAT1-related signals suggests that the observed effect is linked to STAT1-associated pathways. However, LDH can also be influenced by processes beyond hemolysis, and the whole-blood system does not isolate the specific contribution of T cells from that of other blood components; therefore, these ex vivo findings should be interpreted as supportive rather than definitive.

In the AIHA mouse model, treatment with resveratrol or fludarabine reduced erythrocyte-bound IgG, spleen weight, and LDH, and lowered total and phosphorylated STAT1 fluorescence in peripheral blood CD4^+^ and CD8^+^ T cells. Erythrocyte-bound IgG directly reflects RBC-associated antibody binding, a central mechanism in AIHA, and its reduction together with lower LDH and spleen weight supports a treatment-associated improvement in AIHA-related hemolysis. The concurrent decrease in STAT1/pSTAT1 signals in T cells further supports the involvement of STAT1-associated transcriptional regulation in this response (12). These findings are consistent with the proposed role of STAT1 in AIHA pathogenesis and support further mechanistic investigation (13, 14). Nonetheless, neither compound is a selective T-cell STAT1 inhibitor, and these data cannot establish that T-cell-intrinsic STAT1 is the mediator of the observed changes; T-cell-specific genetic perturbation, viability assays, and additional hemolysis readouts would be needed to confirm the mechanism.

Several limitations should be acknowledged. The relatively small sample size and clinical heterogeneity of AIHA may affect the generalizability of our results. All patients in this cohort had primary warm AIHA, which reduced clinical heterogeneity within the study population. However, because of the small sample size and the absence of other AIHA subtypes, the generalizability of these findings to cold agglutinin disease, mixed AIHA, Evans syndrome, secondary AIHA, or other AIHA populations remains limited. Moreover, the *in vitro* whole-blood model, while physiologically relevant, cannot delineate cell-type-specific effects. Future studies using conditional knockout models or single-cell approaches will be essential to definitively establish the causal role of T-cell-intrinsic STAT1 signaling.

## Conclusion

In conclusion, our study integrates clinical observation, transcriptomic profiling, and functional validation to identify STAT1 as a potential regulator associated with AIHA-related T-cell dysfunction. These findings provide a basis for further mechanistic and therapeutic investigation of STAT1-associated signaling in AIHA, while additional studies are needed to establish its causal role.

## Data Availability

The raw data supporting the conclusions of this article will be made available by the authors, without undue reservation.

## Supplementary material

Supplementary Figure 1 Transcription factor prediction via the ChEA3 database for CD4+ T-cell DEGs **(A)**; CD8+ T-cell DEGs **(B)**; and Treg cell DEGs **(C)**.
